# Trichoblastoma and breast carcinoma as metachronous primary tumors: Case report

**DOI:** 10.1016/j.ijscr.2024.110166

**Published:** 2024-08-13

**Authors:** Ahmed A. Almass, Dhuha N. Boumarah, Zhara Al-Ali, Alaa A. Salim, Ahlam A. Dohal, Mohammed Al Duhileb

**Affiliations:** a King Fahd Hospital of the University, Imam Abdulrahman Bin Faisal University, Khobar, Saudi Arabia; b Department of General Surgery, King Fahd Hospital of the University, Imam Abdulrahman Bin Faisal University, Khobar, Saudi Arabia; c Department of Pathology and Laboratory Medicine, King Fahad Specialist Hospital, Dammam, Saudi Arabia; d Department of Radiation Oncology, King Fahad Specialist Hospital, Dammam, Saudi Arabia; e Department of General Surgery, Breast and Endocrine Surgery, King Fahad Specialist Hospital, Dammam, Saudi Arabia

**Keywords:** Multiple primary tumors, Trichoblastoma, Skin adnexal tumor, Breast cancer, Case report

## Abstract

**Introduction and importance:**

The occurrence of more than one tumor originating from the same or different organs is the definition of multiple primary tumors. According to the time of diagnosis, these tumors are classified into two types: metachronous and synchronous tumors. Trichoblastoma is a rare benign skin tumor that is rarely involved in multiple primary tumors, especially in patients with breast cancer.

**Case presentation:**

A 60-year-old male with left breast and lateral chest wall masses. Lastly, he has been diagnosed with invasive ductal carcinoma of the left breast and chest wall trichoblastoma as metachronous primary tumors with no significant genetic background.

**Clinical discussion:**

With the development in the medical field, such tumors are being encountered more. Some authors suggest a relationship between these tumors and genetic mutations. Although rare trichoblastomas can be transformed into malignant tumors and get metastasized.

**Conclusion:**

The diagnosis and management of primary tumors can be challenging in some cases. Researchers should focus on further exploration of the genetic bases and risk factors of such tumors.

## Highlights

• In case of male breast cancer genetic involvement should be checked.
• Trichoblastoma in a patient with breast cancer in generally very rare
• The detection of multiple primary tumors is increasing nowadays.
• Trichoblastoma can be found in the trunk.
• Male breast cancer risk factors are the same as female breast cancer risk factors.

## 1 Background

Multiple primary tumors, including metachronous and synchronous tumors, are defined as the occurrence of more than one tumor originating from the same or different organs. When these tumors are discovered more than 6 months apart, they are called metachronous tumors, on the other hand, when the duration is less than 6 months, they are called synchronous tumors [1]. Cutaneous adnexal tumors are rarely reported among patients with breast cancer. The detection of several tumors existing in a single individual, with trichoblastoma being one of these tumors, is considered generally rare [2]. Trichoblastoma is a rare benign skin adnexal tumor that is commonly seen on the scalp, neck, and face and can mimic basal cell carcinoma, representing a diagnostic challenge in several instances [3]. Herein, we present a case of a 60-year-old male diagnosed with invasive ductal carcinoma of the left breast and chest wall trichoblastoma, along with a comprehensive review of the literature.

The case has been reported in this article in line with Surgical CAse REport (SCARE) 2023 guidelines [4].

## 2 Case presentation

**Fig. 1:**
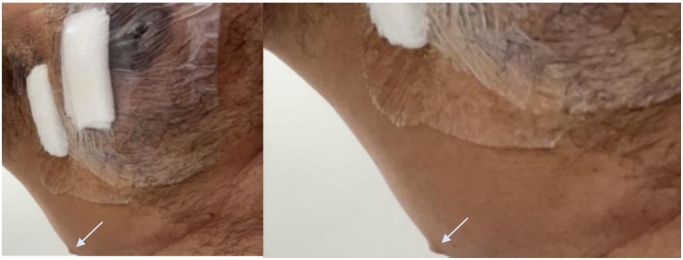
The pointer is a skin lesion (trichoblastoma) that is ipsilateral to the breast which the histopathology biopsy has been taken from.

A 60-year-old Saudi male presented to our breast oncology clinic with a complaint of left breast mass noted 1 year prior to his presentation. The mass was increasing in size and was associated with skin redness. He had been clinically diagnosed with diabetes mellitus type 2 and his past surgical history was only significant for a hemorrhoidectomy performed approximately 20 years ago. He reported no history of hormonal therapy nor radiation exposure and had no family history of malignancies. Upon local examination of the breast, there was a retroareolar mass measuring 6 × 6 cm occupying the whole left breast with nipple ulceration and surrounding peau d'orange appearance of the skin. The left axilla demonstrated a palpable lymph node, hard in consistency and measuring around 1 × 1 cm. The right breast and axilla were unremarkable. The supra and infraclavicular lymph nodes were both negative. On the left lateral part of the chest, a small lesion with a punctum in the center was discovered with a impression of a sebaceous cyst. The patient stated this lesion has been present since 2012 (see Fig. 1).

**Fig. 2:**
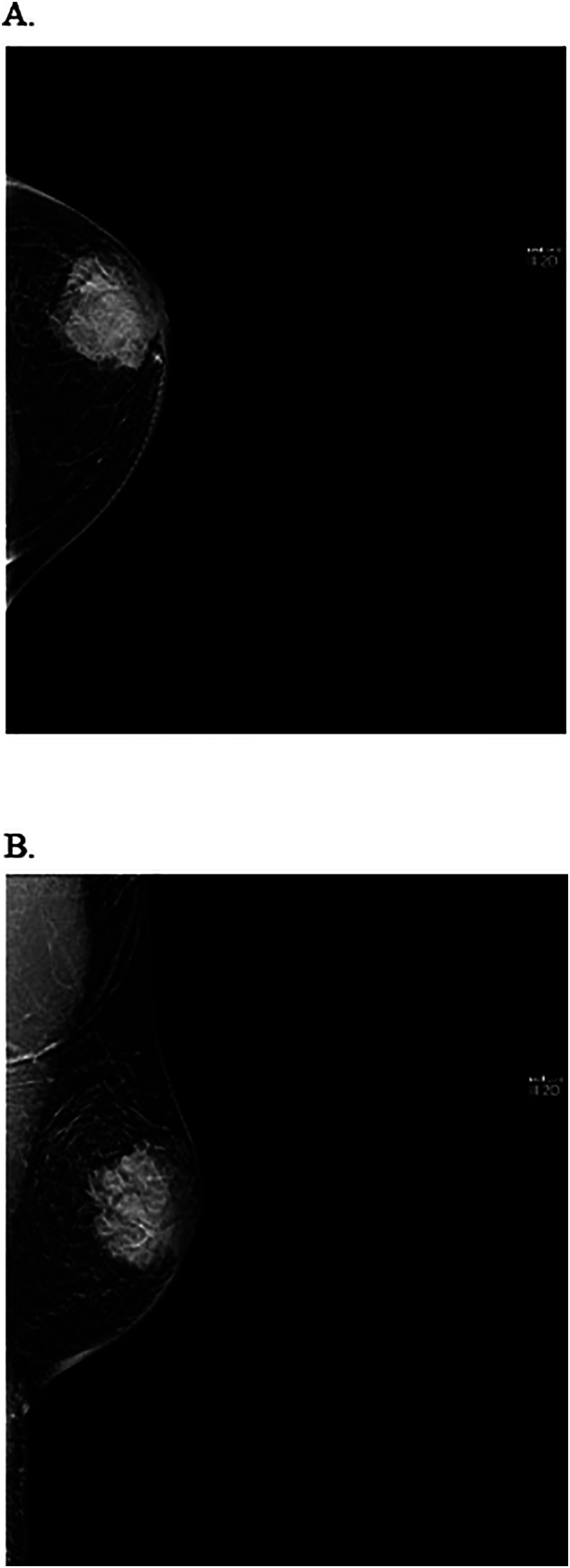
A. Mammogram CC view of the left breast. B. Mammogram MLO view of the left breast showing breast density type A with large lateral retroarealoer hyperdense lesion.

**Fig. 3:**
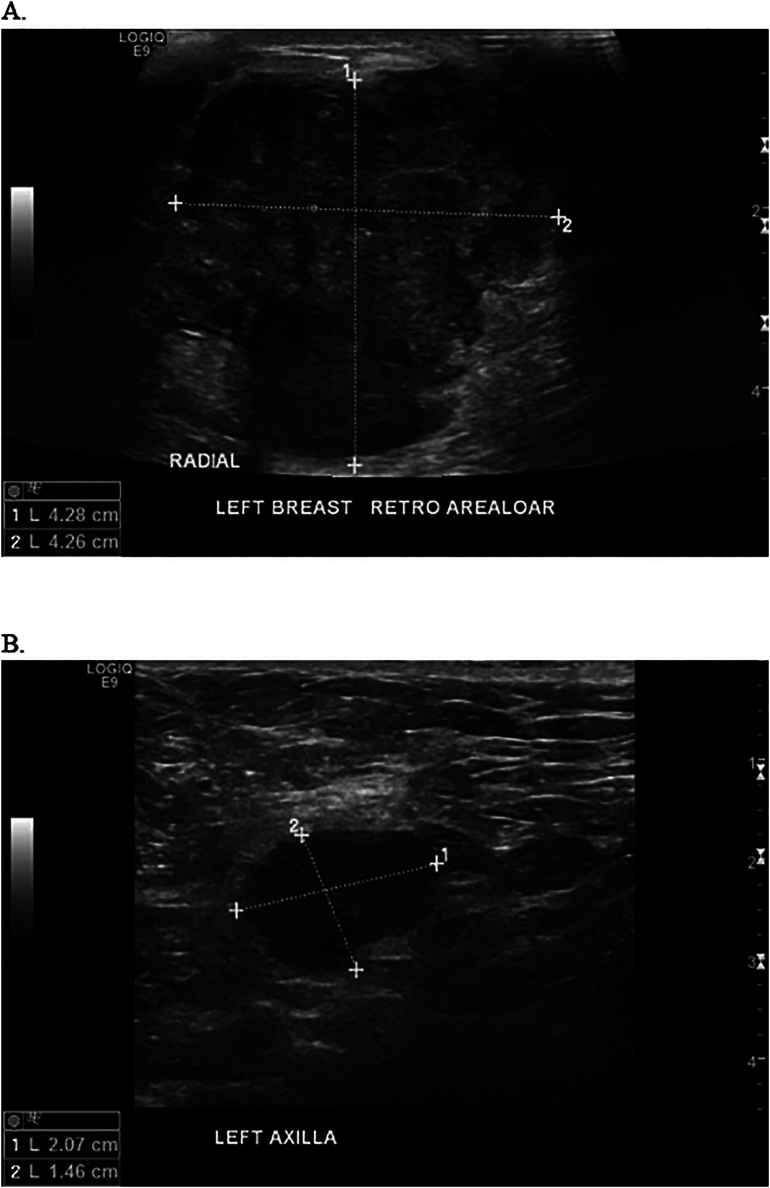
A. Left breast retroarealoer US showing a 4 ∗ 4 CM irregular lesion. B. Left Axilla US showing 2 ∗ 1.5 lymph node with preserve hilum.

**Fig. 4:**
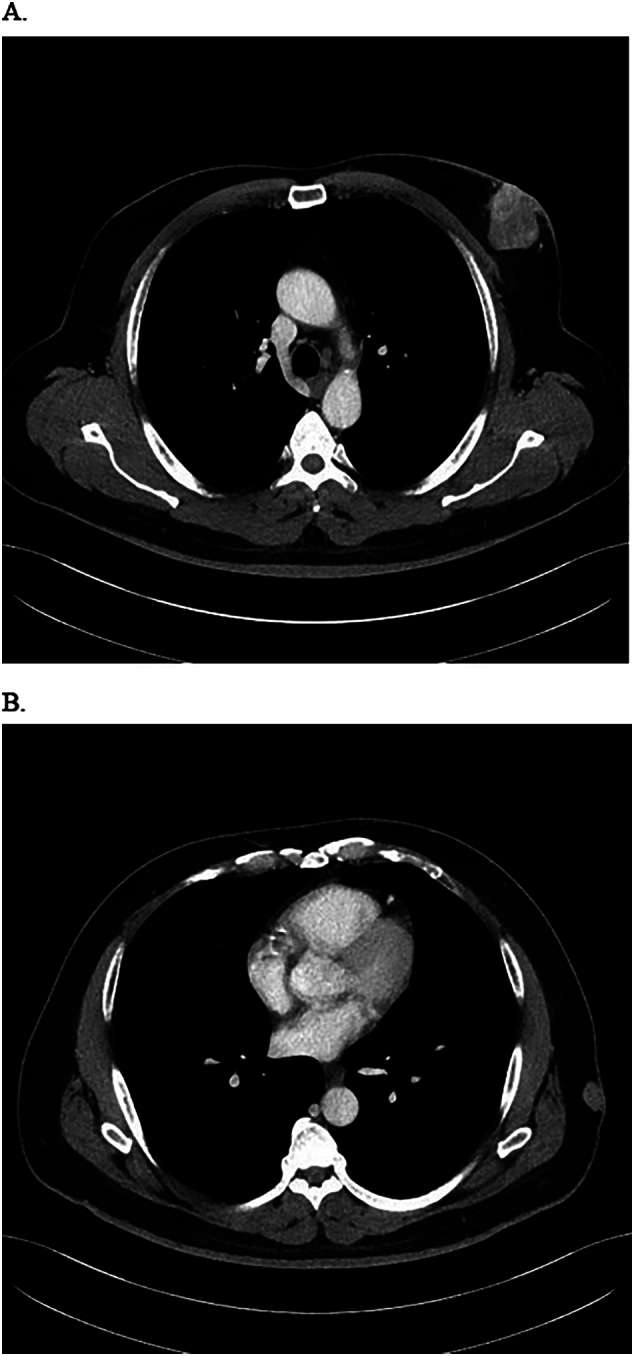
A. Chest CT scan showing left breast hyperdense lesion. B. Chest CT scan showing left lateral chest wall hyperdense lesion.

A diagnostic bilateral breast mammogram and ultrasound (US) showed a 4.3 cm retro-areolar locally advanced left breast mass (irregular with micro-lobulated margins that is inseparable from the nipple associated with skin thickening) and an enlarged left axillary lymph node (1.4 cm) (see Fig. 2, Fig. 3). A computed tomography (CT) scan of the chest, abdomen and pelvis showed no pulmonary or abdominal metastasis yet revealed a subcutaneous enhancing nodule measuring 2.5 × 1.7 cm located over the left lateral chest wall (see Fig. 4). The nodule was initially thought to be a metastatic lymph node. A core biopsy was obtained from the left breast mass and the suspicious left axillary lymph node and illustrated the presence of left breast invasive ductal carcinoma (IDC) grade III, positive for lymphovascular invasion, ER 80 %, PR negative, Her2 negative +1 score and Ki-67 of 30–40 % with positive left-sided lymph nodes, representing a metastatic mammary carcinoma. Given the fact that we were dealing with a male patient, BRCA1 and 2 genetic tests were done, both of which were negative.

**Fig. 5:**
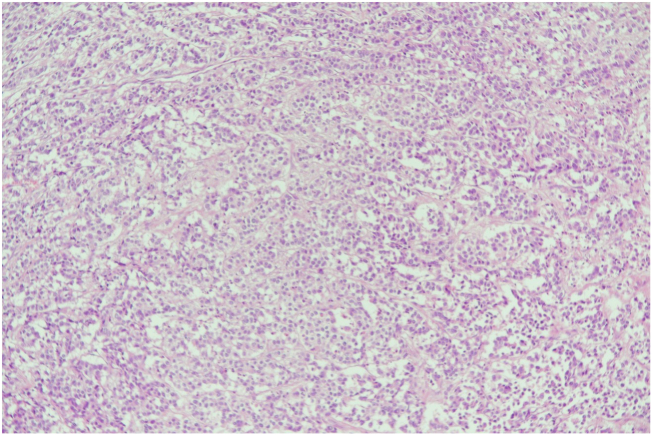
Invasive ductal carcinoma of breast.

**Fig. 6:**
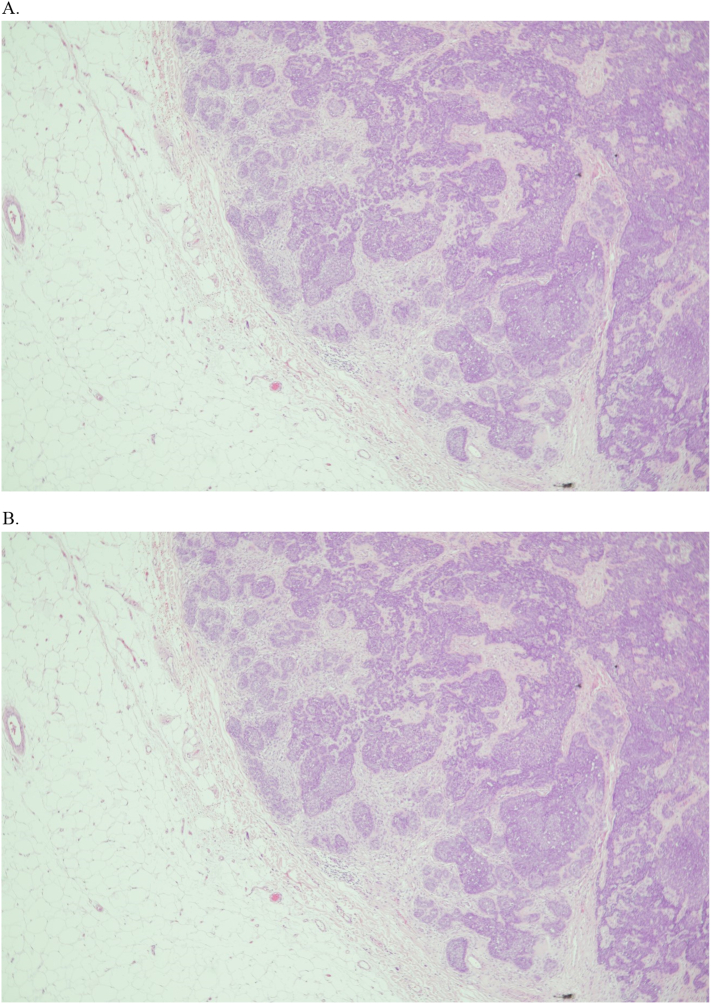
A. Well circumscribed basaloid tumor in the subcutaneous fat shows irregular nests and packed lobules of basaloid cells with variable stromal condensation and pilar differentiation. B. Nests of basaloid cells show peripheral palisading surrounded by fibrous stroma. Clusters of spindled to epithelioid cells are found within the basaloid cell nests.

Our multidisciplinary team agreed to provide him with a left mastectomy and axillary clearance after completing a course of neoadjuvant systemic therapy along with the excision of the left chest wall lesion during the same setting. The final pathology report confirmed the diagnosis of IDC, with negative margins and one positive lymph node for metastasis 1/10 (ypT2N1a) (see Fig. 5). The chest wall nodule was reported as an adnexal skin tumor, specifically trichoblastoma, with the greatest dimension of 2 cm (see Fig. 6). The patient then started to follow up with the oncology and radiation oncology departments for adjuvant treatment.

## 3 Discussion

Invasive ductal carcinoma represents the most common form of breast cancer as it is responsible for approximately 75 % of all breast malignancies [5]. Among all breast cancer cases diagnosed in the United States, less than 1 % were males, additionally, the risk of male breast cancer was estimated to be 1 in 833 compared to 1 in 8 in females [6]. Specifically, around 2800 males were diagnosed with invasive ductal carcinoma (IDC) in 2023 compared to 297,790 females [7]. The entity of multiple primary tumors was first described by Billroth in 1889 who defined it as the occurrence of double or more tumors in one or more organs [8,9]. Although no specific definition has been used to define multiple primary tumors in the literature, it should be ruled out whenever there is possibility that one lesion is a metastasis of the other [1]. Regarding the sub-types of multiple primary tumors, they can be described as synchronous when tumors arise within 6 months or metachronous when they arise within a time interval of more than 6 months [10]. As illustrated in our case, the patient noted the first lesion years prior to the detection of the breast lesion, hence the tumors were labeled as a metachronous in nature.

Cancer survival rates have improved with the enhancement of available diagnostic tests, the availability of highly developed treatment options, and the improvement of screening modalities [1,8]. Interestingly, improvement in survival rates has subsequently increased the detection of multiple primary tumors. The frequency nowadays varies between 2.4 % and 17 % which is attributed to various factors, including patients' awareness [11]. Risk factors of male breast cancer are similar to those noted among females, including family history, obesity, and some genetic syndromes, such as Klinefelter syndrome and Cowden syndrome [12,13]. The incidence of multiple primary tumors in patients with breast cancer ranges from 4.1 % to 16.4 % [1]. According to Luca et al., there are around 12 cases of multiple primary tumors involving the breast and skin, and most of the related skin cancers were melanomas [9].

Trichioblastoma is a benign hair-germ tumor that tends to present as a solitary lesion. Their prevalence has not been thoroughly investigated. Nonetheless, they are commonly seen in patients between 40 and 50 years of age [3,14]. Trichoblastomas rarely metastasize, however, with the loss of p53 tumor suppressor protein and elevation in PI3-AKT signaling pathway, it can transform into trichoblastic carcinoma which carries a risk of local invasion, metastasis, and recurrence [15]. Unlike other adnexal tumors, trichoblastomas were not found to be familial, however, they are the most common tumor associated with nevus sebaceus, and occasionally present on the trunk [3]. The benign nevus sebaceus are skin hamartoma composed of adnexal and epithelial components [16]. As skin adnexal tumors can mimic malignancy and due to the documented transformation sequence, it is recommended to excise these lesions once detected [16,17].

Our search yields a similar case of multiple primary tumors detected in a 71-year-old female who presented with a right breast mass and advised that it had been first noted two years prior to presentation. Following a thorough assessment, she was diagnosed with trichoblastic fibroma of the left breast and breast carcinoma of the contra-lateral side. It is of a clinical and diagnostic significance to note that similar lesions might be mistakenly diagnosed as breast cancer, as they might represent similar features. Shimazaki et al., reported that to differentiate breast cancer from breast trichoblastoma on fine needle aspiration, the basaloid cells with focal squamous eddies, being the composition of the clusters with the presence of focally peripheral palisading, are characteristic features of trichoblastoma by which breast cancer can be excluded [18].

The diagnosis and management of synchronous or metachronous tumors can be challenging regardless of the involved organs. The management of each lesion might compromise the treatment of the other as their indications and contraindications could overlap during the therapeutic course. The question of curative or non-curative intent of treatment might be raised in similar scenarios [1].

## 4 Conclusion

We present a case of chest wall tricoblastoma and ipsilateral breast invasive ductal carcinoma as metachronous tumors detected in a male patient with no significant genetic background. The incidence of several primary tumors is currently increasing with the observed enhancement in cancer survival. As tumors of different origins require various management strategies, each lesion in a patient presenting with multiple tumors should be assessed individually and the possibility of metastasis should be identified. The genetic bases and risk factors of multiple primary tumors need to be further explored.

## Informed consent

Written informed consent was obtained from the patient for publication and any accompanying images.

## Consent

Written informed consent was obtained from the patient for publication and any accompanying images. A copy of the written consent is available for review by the Editor-in-Chief of this journal on request.

## Ethical approval

Ethical approval for this study (IRB- Pub-024-018) was provided by the IRB Committee at King Fahad Specialist Hospital, Dammam, Saudi Arabia on 21 July 2024.

## Funding

This article is not funded by any funding agency.

## Author contribution

1. **Ahmed A. Almass**: study concept or design, data collection, data analysis or interpretation, writing the paper.
2. **Dhuha N. Boumarah**: study concept or design, data analysis or interpretation, writing the paper.
3. **Zhara Al-Ali**: study concept or design, data analysis or interpretation, writing the paper.
4. **Alaa A. Salim**: data collection, data analysis or interpretation.
5. **Ahlam A. Dohal**: data analysis or interpretation.
6. **Mohammed Al Duhileb**: study concept or design, data collection, data analysis or interpretation, writing the paper.

## Guarantor

Ahmed A. Almass.

## Research registration number

This study has been registered in the IRB Committee at King Fahad Specialist Hospital, Dammam, Saudi Arabia on 21 July 2024 with the registration number: IRB- Pub-024-018.

## Conflict of interest statement

The authors declare that they have no competing interests.
